# P-965. Using Simulated Corrective Mentoring to Improve Clinical Decision-Making for Patients with HIV and Identify Drivers for Treatment Choices

**DOI:** 10.1093/ofid/ofae631.1155

**Published:** 2025-01-29

**Authors:** Katie Robinson, Jennifer Frederick, Robert A Esgro, Bharati Hegde, Jennifer Surich

**Affiliations:** Vindico Medical Education, Thorofare, New Jersey; Vindico Medical Education, Thorofare, New Jersey; Vindico Medical Education, Thorofare, New Jersey; Vindico Medical Education, Thorofare, New Jersey; Vindico Medical Education, Thorofare, New Jersey

## Abstract

**Background:**

Due to the vast number of treatment options for the management of patients with HIV, providers are challenged to optimally individualize treatment based on comorbidities, viral resistance, and patient preferences. The use of corrective mentoring in continuing education (CE), is a proven method to improve clinical decision-making while identifying persisting practice challenges.

**Methods:**

Vindico Medical Education and Syandus developed four virtual clinical scenarios designed to encourage optimal decision-making for patients with HIV by providing immediate feedback via simulated corrective mentoring.

**Results:**

As of April 2024, 220 physicians or advanced practice providers involved in the management of patients with HIV had participated in the simulations. Across simulations, only 27% of initial decisions were optimal. At baseline, the most challenging topics regarding medication management were related to management during pregnancy (33% optimal), initial ART selection (49% optimal), and identifying candidates for injectable therapy (44% optimal). However, after corrective mentoring, there was over a 300% increase in optimal decisions made. Of optimal decisions, 48% of clinicians preferred BIC/FTC/TAF as initial treatment selection for a young male with no comorbidities and 47% preferred this regimen for an older patient with early-stage chronic kidney disease (CKD) and various other cardiovascular comorbidities. However, for a patient who is pregnant, BIC/FTC/TAF was also the preferred regimen, even though at the time of the simulation development there was insufficient data to recommend its use. These findings suggest that comfortability with certain regimens may be a driving force for clinical decision-making, even when those decisions may not be ideal. Drivers of choosing alternatives such as CAB/RPV or DTC/3TC include avoidance of weight gain, reduced pill burden, and later stage CKD.

**Conclusion:**

The use of corrective mentoring in simulation-based CE is an impactful way to encourage clinical decision-making aligned with the latest evidence. Moreover, these data highlight the need to educate clinicians who manage patients with HIV on the importance of individualizing treatment based on patient characteristics.

**Disclosures:**

**All Authors**: No reported disclosures

